# *C*. *elegans* germline cell death, live!

**DOI:** 10.1371/journal.pgen.1007425

**Published:** 2018-07-19

**Authors:** Gabriela Huelgas Morales, David Greenstein

**Affiliations:** Department of Genetics, Cell Biology and Development, University of Minnesota, Minneapolis, Minnesota, United States of America; University of California San Diego, UNITED STATES

*“To be*, *or not to be*: *that is the question*: *Whether 'tis nobler in the mind to suffer the slings and arrows of outrageous fortune*, *or to take arms against a sea of troubles*, *and by opposing end them*.*”—Shakespeare*, Hamlet, *Act 3*, *Scene 1*.

Ironically, oogenesis, the developmental process that generates the female gamete, the major conveyor of germline immortality across generations, involves a fateful decision—to commit suicide or to complete development. In organisms as diverse as worms, flies, and mice, oocyte precursors, oogonia, are generated in excess of the number of fully grown oocytes produced for fertilization. This surfeit is culled by apoptosis, also called programmed cell death [1–3], which is mediated by the core apoptotic machinery comprised of CED-9/Bcl2, CED-4/Apaf-1, and the CED-3/Caspase [4]. Apoptosis in the female germline has been proposed to play two major functions: a nurse cell function, in which the oogonia chosen for apoptosis provide their cytoplasm and organelles to other developing oocytes, and a quality control function, in which cells containing a damaged genome or dysfunctional organelles (especially mitochondria) are culled.

**Fig 1:**
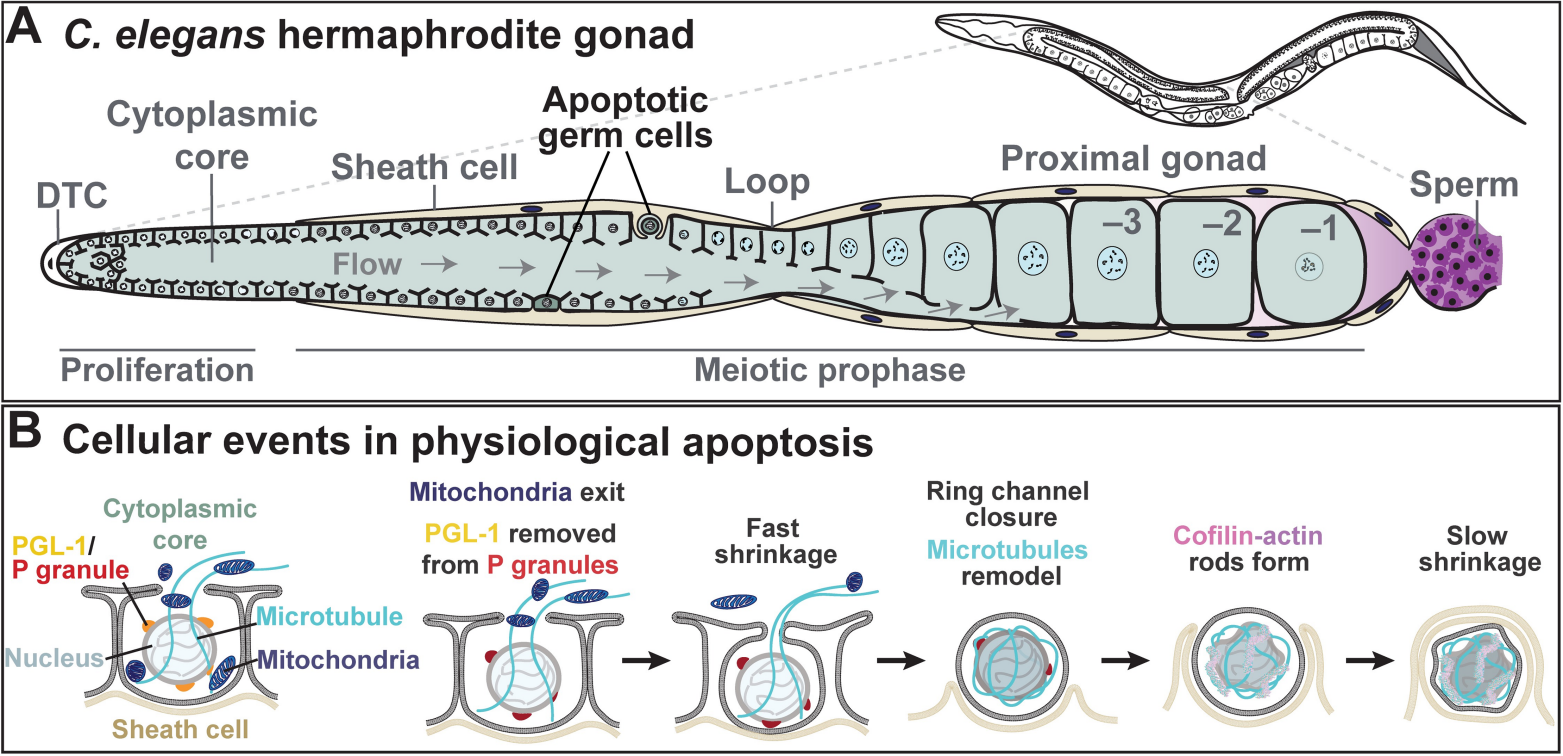
Apoptosis in the *C*. *elegans* germline. *C*. *elegans* hermaphrodite gonad (A) and the progression of cellular events during physiological apoptosis in germ cells (B), as described by Raiders and colleagues. DTC, distal tip cell.

In *Caenorhabditis elegans*, more than half of all female germ cells undergo apoptosis [1,5]. A key unanswered question in the field is how female germ cells are chosen to adopt the apoptotic fate in the correct numbers. This issue has been challenging to address because the characteristic refractile appearance of apoptotic germ cells corresponds to a late stage in the process occurring during or after the engulfment of the dying germ cell by neighboring somatic gonadal cells, the sheath cells (Fig 1A). The paper by Raiders and colleagues in this issue of *PLOS Genetics* reports a tour-de-force analysis of early events in the cell biology of germline apoptosis using multiple microscopic modalities, including live-cell imaging conducted in the wild-type and engulfment-defective mutants [6]. The breathtaking images and videos give us our earliest views into the execution of the apoptotic program in female germ cells and provide strong evidence that apoptosis serves both a nurse cell function and a quality control mechanism.

Raiders and colleagues used a combination of live imaging and an examination of fixed samples by fluorescence and transmission electron microscopy (TEM) to elucidate cellular events occurring during the execution stage of the apoptotic program. They noticed that dying germ cells were devoid of mitochondria. Using live-cell imaging, they observed the selective export of mitochondria from doomed germ cells into the cytoplasmic core of the germline (Fig 1B). Mitochondrial export is dependent on CED-3/Caspase activity and represents the earliest described cellular event in germline apoptosis. Mitochondrial export appears to occur along radial microtubules extending through the ring channels and requires the plus-end–directed motor kinesin, consisting of the UNC-119 kinesin heavy chain and the KLC-1 kinesin light chain. The authors show that the mitochondrial Rho GTPase (Miro), which is involved in mitochondrial transport in neurons [7], is not required for selective mitochondrial export. Thus, this mitochondrial export occurs by a new mechanism. Mitochondria are maternally inherited in all but a few animal species. Evidence suggests that a process of purifying selection operates during oogenesis to ensure the inheritance of maximally fit mitochondria and to protect against mitochondrial bottlenecks during germline development [8]. Interestingly, Raiders and colleagues noticed that, in a few instances, apoptotic germ cells contained one mitochondrion or a few clumped mitochondria. It is tempting to speculate that selective mitochondrial export functions as part of the purifying selection process by recycling the most active mitochondria into the germline pool but relegating inactive or damaged mitochondria to the dust heap with the dying germ cell corpse. Further work will be needed to test this hypothesis and unravel the molecular mechanisms of selective mitochondrial export.

During apoptotic execution, Raiders and colleagues observed two stages of cell shrinkage. The first, referred to as “fast shrinkage,” commences approximately 80 min before the dying germ cell exhibits optical refractility. By contrast, “slow shrinkage” occurs following the closure of the ring channels (Fig 1B). During fast shrinkage, PGL-1, a defining component of perinuclear ribonucleoprotein particles called “P granules,” which are associated with nuclear pore complexes and represent the primary sites of mRNA export to support oogenesis [9], disappears from the undone germ cells. Despite the loss of PGL-1, P granules were observed by TEM until the final stages of apoptosis. Whether the loss of PGL-1 and perhaps associated P granule components reflects their trafficking back to the cytoplasmic core for reuse remains to be determined. Nonetheless, the slow shrinkage and the selective export of mitochondria likely represent the final gasp of the dying cell in fulfilling its role as a nurse cell, which is terminated upon the closure of the ring channels (Fig 1B).

Upon closure of the ring channels, the dying cell extensively remodels its cytoskeleton. Microtubule “cocoons,” closely apposed to the cell cortex and the nucleus, form rings in the apoptotic cells soon after ring channel closure. The apoptotic cells also extensively reorganize their actin cytoskeletons by forming large cofilin-actin rods approximately 40–70 min after ring channel closure. Interestingly, cofilin-actin rods have been observed in several neurodegenerative diseases including Alzheimers and Huntingtons disease. Thus, not only does the dying cell actively participate in its own demise, but it erects cytoskeletal elements that are defining features of the process. These cytoskeletal changes may reinforce the structure of the dying cell to protect the germline or the engulfing cell from degradative activities, including activated caspases.

An unexpected finding of the study is that binucleate and trinucleate germ cells appear to form as byproducts of morphogenesis of the syncytium. These aberrant multinucleate germ cells are eliminated by apoptosis. This observation documents a clear role for germline apoptosis in serving a quality control mechanism. The mechanisms that surveil and recognize multinucleate germ cells and trigger CED-3 caspase activation remain to be determined.

The work of Raiders and colleagues illustrates the continued power of descriptive studies in developmental biology. New microfluidic methods may enable longer term observation of the apoptotic process [10]. These invaluable insights and tools will help researchers solve the mystery of how germ cells make their fateful choice between life and death.
